# The human 18S rRNA m6A methyltransferase METTL5 promotes tumorigenesis via DEPDC1 in lung squamous cell carcinoma

**DOI:** 10.3389/fonc.2025.1522157

**Published:** 2025-02-13

**Authors:** Yang Yan, Jianjun Fu

**Affiliations:** Department of Cardiothoracic Surgery, Gaoxin Branch of The First Affiliated Hospital, Jiangxi Medical College, Nanchang University, Nanchang, China

**Keywords:** LUSC, METTL5, DEPDC1, biomarker, m6A modification

## Abstract

**Background:**

N6-Methyladenosine (m6A) is one of the post-transcriptional modifications and abnormal m6A is critical for cancer initiation, progression, metastasis in Lung squamous cell carcinoma (LUSC). Ribosomal RNA (rRNA) accounts for most of the total cellular RNA, however, the functions and molecular mechanisms underlying rRNA modifications in LUSC remained largely unclear.

**Methods:**

High-throughput library screening identifies the key m6A regulator METTL5 in LUSC. Cell and animal experiments were used to identify that METTL5 promoted LUSC tumorigenesis to enhance DEP domain containing 1 (DEPDC1) translation via m6A modification.

**Results:**

We showed that the N6-methyladenosine (m6A) methyltransferase METTL5 was an independent risk factor in LUSC and was associated with poor prognosis of patients. Notedly, overexpression METTL5 promoted LUSC tumorigenesis in an m6A modification, while METTL5 knockdown markedly inhibited proliferation and migratory ability of tumor cells *in vitro* and *in vivo*. Mechanistically, METTL5 promoted LUSC tumorigenesis via m6a methyltransferase to increase the translation of DEPDC1.

**Conclusion:**

Our results revealed that METTL5 enhances DEPDC1 translation to contribute to tumorigenesis and poor prognosis, providing a potential prognostic biomarker and therapeutic target for LUSC.

## Introduction

Lung cancer is the most common cancer and the highest mortality in the world (1). According to the histological characteristics, lung cancers are classified into small-cell lung carcinoma (SCLC) and non-small cell lung carcinoma (NSCLC) including lung adenocarcinoma (LUAD), lung squamous cell carcinoma (LUSC), and large cell carcinomas (2). Previous studies have indicated the complex multiple altered genes and pathways (3). Although targeted therapy and immunotherapy have improved significantly recently, the efficacy is still far from ideal. Hence, it is an urgent need to further illustrate the molecular pathogenesis of LUSC to develop new, effective therapeutic targets.

Emerging evidence has demonstrated that N 6‐methyladenosine (m6A) modification of RNA play a crucial role in the gene expression of multiple cancers (4). The m6A modifications are dynamic and reversible posttranscriptional RNA modifications in messengerRNA (mRNA), transferRNA (tRNA), and ribosomalRNA (rRNA) that are mediated by three types of effector proteins: writers (“methyltransferases”: METTL3, METTL5, METTL14, and WTAP, erasers (“demethylases”: ALKBH5 and FTO), and readers (“m6A-binding proteins”: YTHDs and IGF2BPs), which regulates RNA splicing and influences the stability and translation of modified RNAs (5).

Importantly, Methyltransferase 5, N6‐adenosine (METTL5) is an 18S rRNA methyltransferase that increases protein translation activity (6). Such as, METTL5 stabilizes c-Myc to reprogram glucose metabolism in hepatocellular carcinoma (7). METTL5 promotes oncogenic mRNA translation via 18S rRNA m6A modification (8). However, the role of METTL5 m6A writer protein in the progression of LUSC remain poorly understood.

DEPDC1 is a highly conserved protein in several species, including Caenorhabditis elegans and mammals, and DEPDC1 is mainly expressed in testis and minimal expression in other normal human tissues (9, 10). Previous studies have demonstrated that DEPDC1 is involved in the malignant progression of multiple tumors, including non-small cell lung cancer (NSCLC) (11), hepatocellular carcinoma (12).

Here, we demonstrated that METTL5 enhances DEPDC1 translation via m6A modification to facilitate LUSC carcinoma progression. In summary, our results revealed that METTL5 is a potential therapeutic target and prognostic biomarker for LUSC.

## Materials and methods

### Data collection

RNA-sequence (RNA-seq) of METTL5 knockout (KO) were downloaded from GEO data (GSE174420), the cut-off criteria were set as | log2 fold change (FC) | < -0.5 and p < 0.05. RNA-sequence (RNA-seq) transcriptional data and clinical information of LUSC were downloaded from the Cancer Genome Atlas (TCGA) database (https://cancergenome.nih.gov/).

### Differentially expressed genes analysis

Differentially expressed genes (DEGs) between high- and low- METTL5 expression groups were analyzed in the TCGA datasets using the “limma” package of R software. The cutoff criteria were set as | log2 fold change (FC) | > 0.5 and p < 0.05. DEGs between normal and tumor tissues were selected based on the Wilcoxon rank test.

### Construction of a protein-protein interaction network

The mRNAs were included in a PPI network using the STRING database (https://string-db.org/) with a confidence score of > 0.8. Cytoscape (version 3.8.1) was used to visualize the PPI network (13).

### Gene set variation analysis

The variation in biological processes between low- and high-METTL5 groups were performed by GSVA analysis using the R package ‘GSVA’.

### Survival analysis

Kaplan-Meier survival analysis was used to evaluate the difference in overall survival (OS) between the two groups using the log-rank test.

### Cell culture and cell culture

Human LUSC cells lines (SK-MES-1 and NCI-H226) were obtained from the American Type Culture Collection and cultured in L-15 medium containing 10% fetal bovine serum (Gibco) at 5% CO2 and 37°C.

### CCK-8 assay, colony formation assay, wound healing assay, and transwell migratory experiment

For the CCK-8 assay, LUSC cells (2 × 10^3^ cells, 100 μL per well) were seeded into 96-well plates, and then CCK-8 reagent was used to assess tumor cells viability following the manufacturer’s instructions at 24, 48, 72, and 96 hours. The absorbance at 450 nm was measured using a microplate reader.

For the colony formation assay, cells were seeded in 6-well plates at a density of 1 × 10^3^ cells per well and cultured for the 7-10 days. The colonies were then stained with 0.2% crystal violet and documented.

For the wound healing assay, Cells were seeded in 6-well plate and incubated overnight. Next day, the inserts were removed, and serum-free medium was added. After 36 h. ImageJ was used to calculate the migratory rate.

For the transwell migratory experiment. To evaluate cell migratory ability, tumor cells (5 × 10^4^ cells) were seeded into the upper chamber of Transwell inserts. The lower chamber was filled with 500 μL of complete medium containing 10% FBS to induce cell migration. After 24 hours of incubation, cells that migrated through the membrane were stained with 0.2% crystal violet and photographed by microscopy.

### Western blot

We extract total protein from cells using RIPA buffer (Beyotime, Shanghai), then the BCA kit (Beyotime, China) was used to detect the protein concentration. We separated lysates by utilizing 10% SDS–PAGE and transferred onto polyvinylidene difluoride (PVDF) membranes. Membranes were incubated overnight at 4°C with primary antibodies. After washing, membranes were incubated with the corresponding secondary antibodies at room temperature for 2 hours.

### Chemical reagents, antibodies, and transfection

Anti‐METTL5 (proteintech, China, Cat# 16791-1-AP); DEPDC1 (bs-6525R, Bioss); METTL5 and DEPDC1 overexpression plasmids and short hairpin RNA (shRNA) METTL5 (**Supplementary Table S4**) were purchased from GeneChem (Shanghai, China). All transfections were performed according to the manufacturers’ instructions.

### Quantitative RT–PCR

Total RNA was extracted from cells using TRIzol Reagent (ThermoFisher, Waltham, MA, USA) according to the manufacturer’s instructions. 1 μg of RNA was applied for reverse transcription (Cat. #R323-01, Vazyme). qRT‐PCR reactions were carried out using the ABI 7500 system (Applied Biosystems; Foster City, CA, USA). The amplification primers were listed in **Supplementary Table S5**.

### Analysis of modified nucleosides by liquid chromatography‐mass spectrometry

The 18S rRNAs and mRNA were purified from cells by 10–50% sucrose velocity centrifugation. Briefly, 1 × 107 cells were seeded and detached in PBS at 0°C and resuspended in 500 μl Buffer. The cell suspension was centrifuged at 20 000 × g for 20 min at 4°C, and extracts were thawed on ice and loaded on a 10–50% sucrose density gradient and centrifuged at 23 000 × g for 20 h in a Beckman L-90K centrifuge with a SW41Ti rotor. RNA was extracted from peak fractions with TRIreagent (Sigma #T9424). LC/MS was used to analyze the m6A levels of 18S rRNAs and mRNA (7).

### Dual‐luciferase assay

Luciferase reporter expression was performed using the Dual Luciferase Assay Kit (Promega, Madison, WI, USA). METTL5‐WT and METTL5‐KD were transfected into SK-MES-1 and NCI-H226 cells using Lipofectamine 3000 (Thermo Fisher, Carlsbad, CA, USA) according to the manufacturer’s instructions. The results were normalized for transfection efficiency using Renilla luciferase activity.

### Polysome profiling

Briefly, cells were incubated with Cycloheximide (CHX; Sigma–Aldrich) for 5 min at 37°C, and then the cells were washed with PBS containing 100 μg/mL CHX after removing the medium. Next, 400 μL of Triton X‐100‐containing lysis buffer were added and incubated with the cells for 15 min. Each cell suspension was centrifuged, and the supernatant was collected. Subsequently, a 10%‐50% sucrose gradient was prepared in lysis buffer without Triton X‐100. Cell lysates were loaded onto a sucrose gradient and centrifuged at 30000 rpm for 4h at 4°C. The samples were then fractionated and analyzed with a Gradient Station.

### Tumor xenograft model

Male BALB/c nude mice (4–6 weeks, 18–22 g) purchased from the animal center of Biotechnology Co., Ltd (Beijing, China) were maintained under specific pathogen-free conditions. Treated Huh-7 cell suspensions (1 × 10^6^ cells) were mixed 1:1 and injected subcutaneously into the right axillae of nude mice. Quantification of immunohistochemical staining was performed using Image-Pro Plus 6.0 software. Tumor volume was calculated as follows: (longest diameter) × (shortest diameter)2 × (π/6). All animal experimental procedures used in this study were approved by the Animal Ethics Committee of Gaoxin Branch of The First Affiliated Hospital, Jiangxi Medical College, Nanchang University.

### Statistical analysis

Statistical analyses were performed using R, version 4.2.3. For comparisons, Student’s t-test and Wilcoxon test were applied to compare the differences between groups; one-way ANOVA was used for comparisons among three or more groups. Correlations were analyzed by using Pearson’s correlation. Univariate and multivariable survival analysis were performed using Cox regression analysis. Survival analysis was performed by the Kaplan–Meier method with a log rank test. All experiments were performed for at least 3 times independently under similar conditions, unless otherwise specified in the figure captions. In all cases, p value < 0.05 was considered statistically significant.

## Results

### High-throughput library screening identifies METTL5 as a core m6A regulator in LUSC

**Figure 1:**
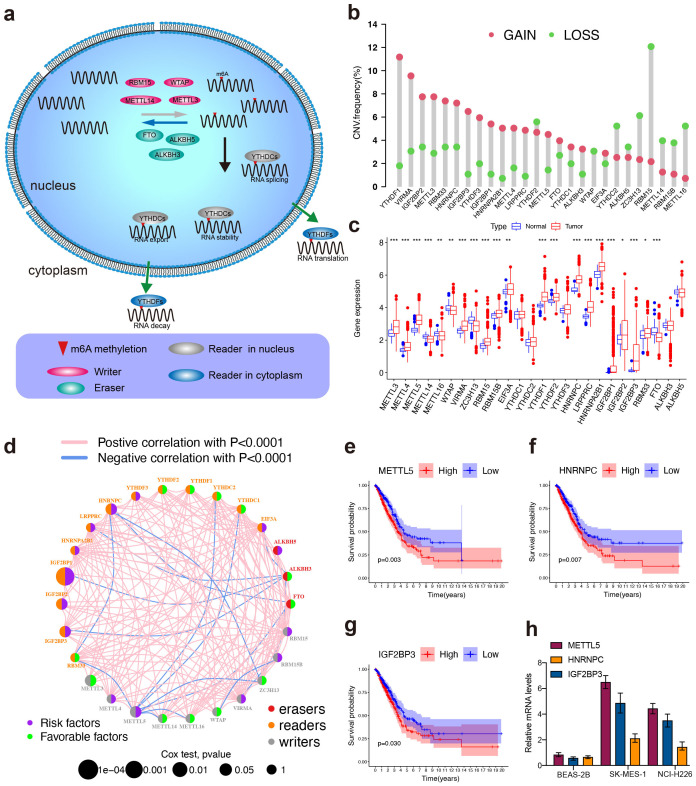
High-throughput library screening identifies the key m6A regulator in LUSC. **(A)** Mechanism of RNA m6A Modifications. **(B)** The CNV variation frequency of m6A regulators in TCGA cohort. The height of the column represented the alteration frequency. The amplification frequency, red dot; the deletion frequency, green dot. **(C)** Differential expression of m6a regulators between normal and LUSC tissues in TCGA cohort. (Normal: blue and LUSC: red). Significant results are indicated as ***p < 0.001, **p < 0.01, and *p < 0.05. **(D)** The interaction of expression on m6A regulators in LUSC. Different biological functions of m6A regulators were depicted by circles in different colors. The circle size represented the effect of each regulator on the prognosis by P-value. The lines linking regulators showed their interactions, pink represented positive correlation, and blue represented negative correlation. Green dots in the circle showed favorable factors of prognosis. Purple dots in the circle showed risk factors of prognosis. **(E–G)** Kaplan–Meier survival analysis showed that indicated genes (METTL5, IGF2BP3, and HNRNPC) exhibited prognosis in LUSC patients based on TCGA data. **(H)** mRNA expression levels of METTL5, IGF2BP3, and HNRNPC were detected using pPCR in SK-MES-1 and NCI-H226.

The process of N 6‐methyladenosine (m6A) modification of RNA was dynamically and reversibly regulated by these m6A regulators (**Figure 1A**, **Supplementary Table S1**). A total of 26 m6A regulators including 10 writers, 3 erasers and 13 readers were performed for subsequent studies based on TCGA data. The investigation of CNV alteration frequency showed a prevalent CNV alteration in 26 regulators and most were focused on the amplification in copy number. YTHDF1 and VIRMA showed amplification frequencies, whereas ZC3H13 and RBM15 had CNV copy number deletions (**Figure 1B**). Compared to normal tissues, m6A regulators with amplificated CNV demonstrated remarkedly higher expression in LUSC tissues, and vice versa (**Figures 1B, C**). The comprehensive landscape of m6A regulator interactions, regulator connection and their prognostic significance for LUSC patients was depicted with the m6A regulator network (**Figure 1D**). We found that not only exhibited significant correlations in expression within the same functional category but also among writers, erasers, and readers. Importantly, based on risk and survival analysis, the 3 best hints, METTL5, HNRNPC, and IGF2BP3 (**Figures 1E–G**), were screen, of which METTL5 mRNA expression was the highest in LUSC cells lines (SK-MES-1 and NCI-H226) (**Figure 1H**). Taken together, these data highlight the oncogenic role of METTL5 in LUSC progression.

### METTL5 is an independent prognostic factor in LUSC

**Figure 2:**
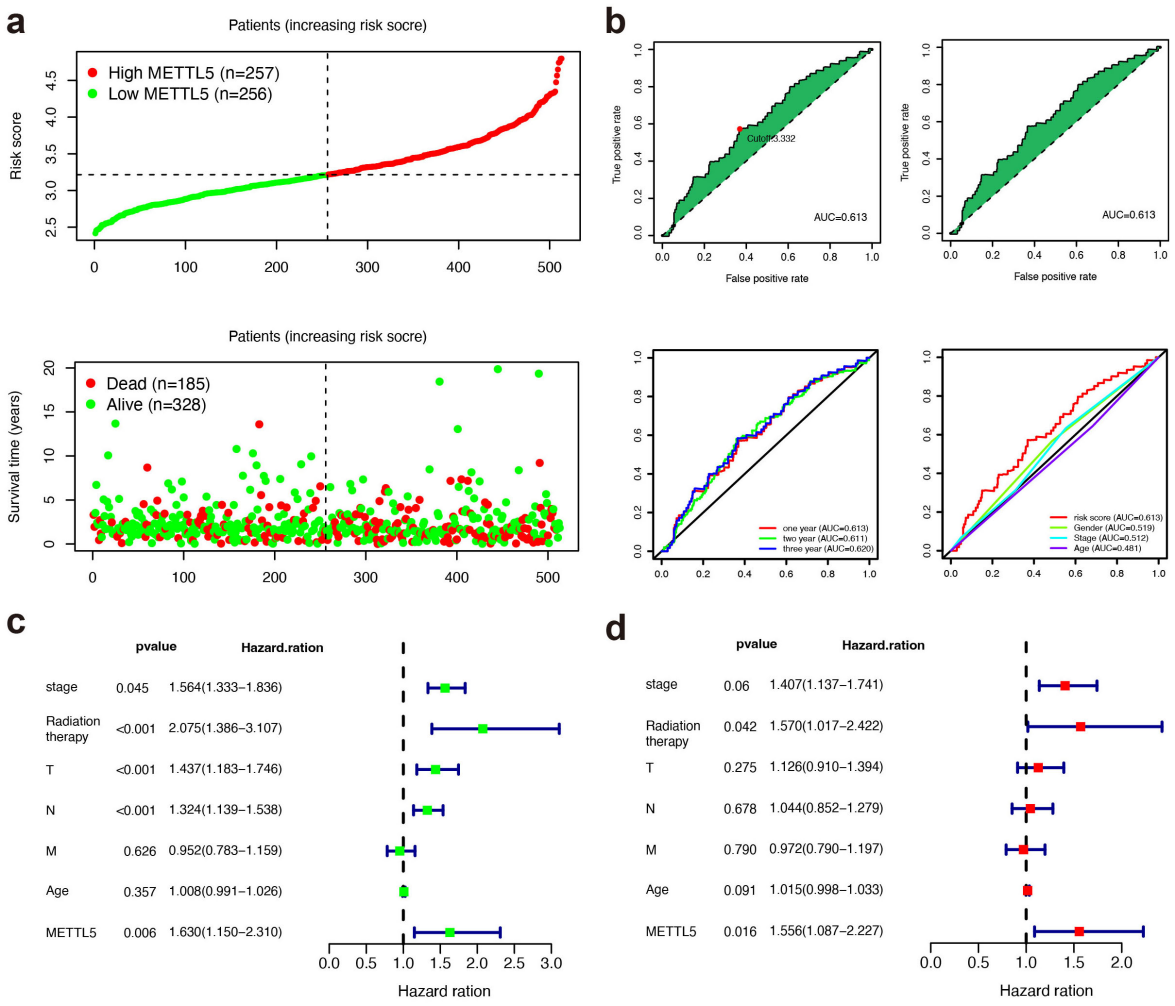
METTL5 is an independent prognostic factor in LUSC. **(A)** Distribution of risk score and OS status in the high- and low-METTL5 expression subgroups. **(B)** The AUC value and cutoff point obtained in the TCGA set, ROC curve analysis within 0.5, 1, and 1.5 years, and multivariate ROC curve analysis showing that the superior prognostic performance of the METTL5 expression compared to other clinical indicators. **(C, D)** Univariate analysis **(C)** and multivariate analysis **(D)** of the METTL5 expression and other clinical information in TCGA LUSC cohort.

Based on the TCGA dataset, high METTL5 expression was related with higher risk score and poorer OS status in LUSC patients (**Figure 2A**). The areas under the curves (AUCs) for 0.5-, 1-, and 1.5-year OS were 0.613, 0.611, and 0.620, respectively using the receiver operating characteristics (ROC) analysis, which showing the predictive ability for METTL5 (**Figure 2B**). In univariate Cox analysis revealed that METTL5 was an independent prognostic factor (**Figure 2C**). In multivariate Cox analysis, the METTL5 was significantly better than those of other clinicopathological indicators (**Figure 2D**). Together, these data illustrate METTL5 is an independent prognostic factor in LUSC.

### METTL5 promotes tumor progression in LUSC

**Figure 3:**
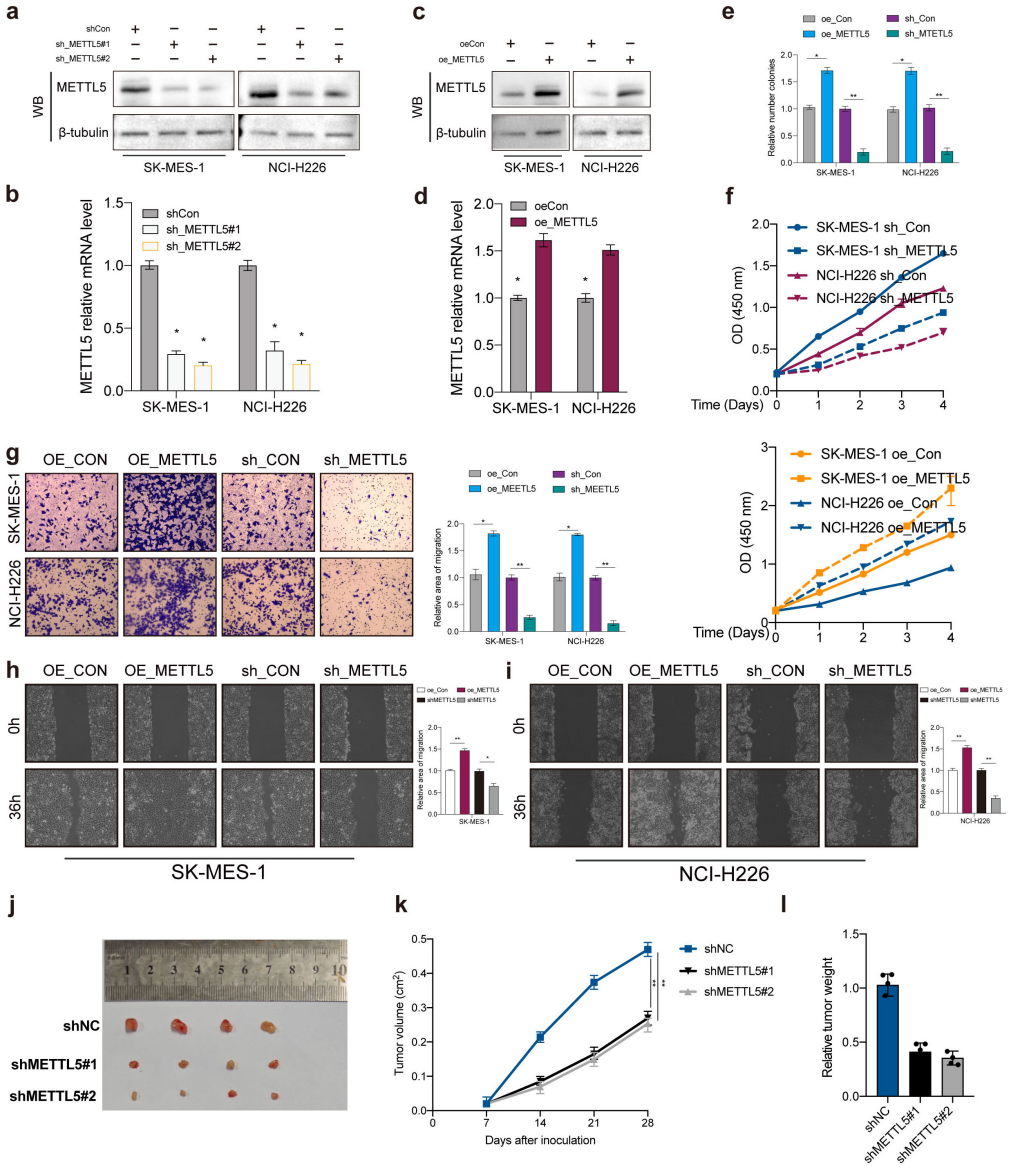
METTL5 promotes tumor progression in LUSC. **(A–D)** Transfection knockdown or overexpression efficiencies was validated by western blot and qPCR, respectively. **(E, F)** Colony formation assay and CCK-8 was used to analyze the proliferation viability of METTL5 in LUSC cell. All the data are presented as the mean ± standard deviation (n = 3). *P < 0.05, **P < 0.01, compared with the control group. **(G–I)** Transwell migration assay and wound healing assay was used to analyze the migration viability of METTL5 in LUSC cell. All the data are presented as the mean ± standard deviation (n = 3). *P < 0.05, **P < 0.01, compared with the control group. **(J)** The image of mice bearing subcutaneous tumors derived from SK-MES-1 cells treated with different treatment (shNC, shMETTL5#1, or shMETTL5#2) at the indicated times. **(K)** The xenograft growth curves for the shMETTL5#1, shMETTL5#2, and shNC groups were plotted by measuring the tumor size (width2 × length × π/6) with a Vernier caliper every 7 days. **(L)** Nude mice were sacrificed, and xenografts were harvested and weighed.

To explore the biological function of METTL5 in LUSC, we transfected METTL5 knockdown or overexpression in SK-MES-1 and NCI-H226 cells. Transfection efficiency was evaluated by western blot and qPCR (**Figures 3A–D**). Colony formation assays and CCK-8 showed that METTL5 overexpression promoted cell proliferation and colony formation ability, whereas METTL5 knockdown decreased those (**Figures 3E, F**). In addition, our research verified that METTL5 also regulated the migration and invasion capabilities of LUSC cells (SK-MES-1 and NCI-H226 cells) (**Figures 3G–I**). The SK-MES-1 cell suspension mixtures were used to establish an *in vivo* xenograft model. During the observation of flank xenografts in BALB/c nude mice for 28 days, METTL5 knockdown significantly decreased tumor volume compared with that of control tumors (**Figures 3J, K**). The nude mice were sacrificed, and the xenografts were harvested and weighed, the results showed tumor weights was significantly reduced (**Figure 3L**). Therefore, these results suggested that METTL5 promotes tumor progression in LUSC.

### Functional annotations of METTL5 in LUSC

**Figure 4:**
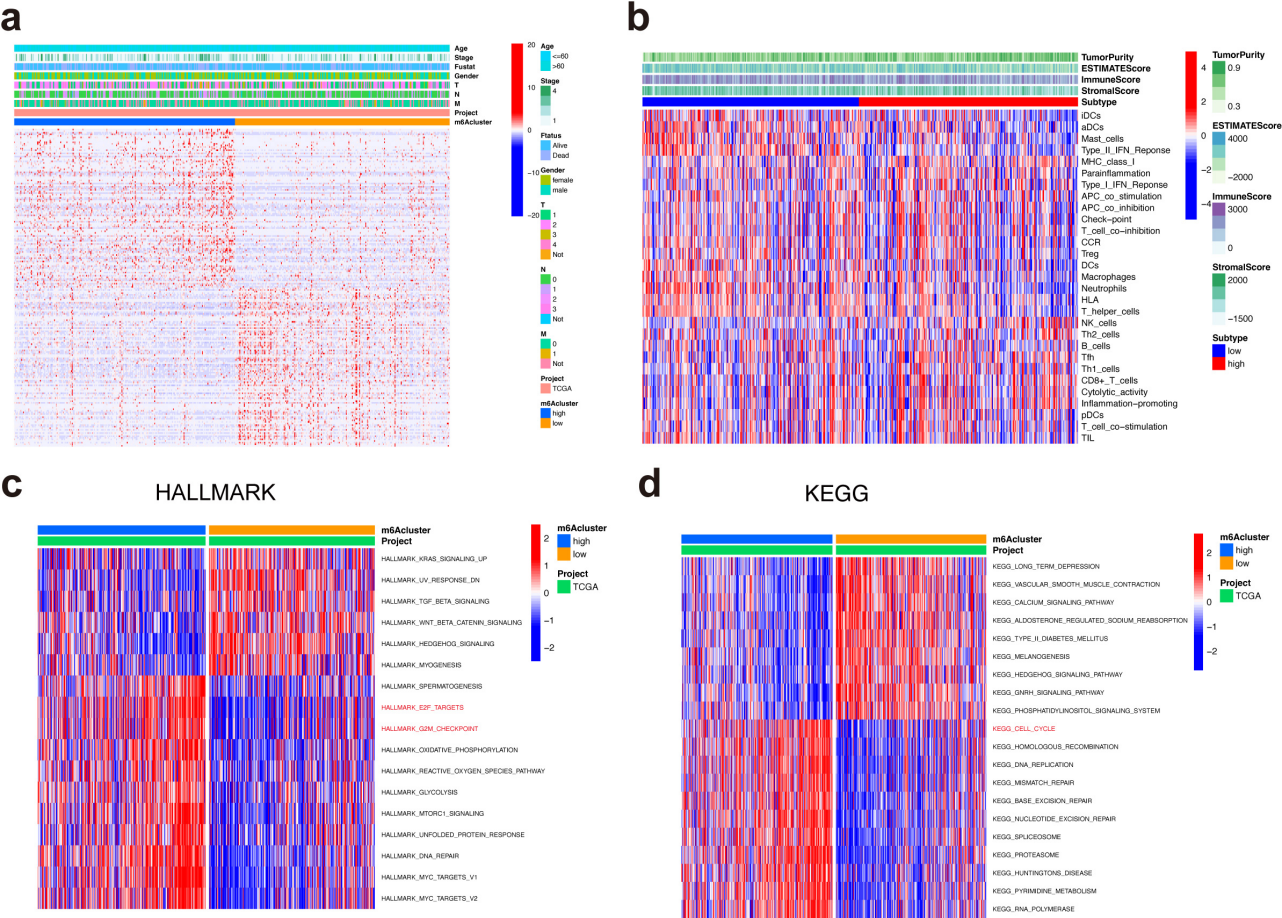
Functional annotations of METTL5 in LUSC. **(A)** Associations between METTL5 expression and clinical features of LUSC. **(B)** Correlations between METTL5 expression and immune-interrelated signatures, as determined by ESTIMATE, immune, stromal, and tumor purity scores. **(C)** GSVA analyses (HALLMARK) of METTL5 in LUSC patients. **(D)** GSVA analyses (KEGG) of METTL5 in LUSC patients.

The correlations between METTL5 expression and clinical properties were evaluated in the TCGA dataset, we found that METTL5 expression level was positive with older age, gender, and tumor stage (**Figure 4A**). Immune cell infiltration analysis showed that high-METTL5 patients were more increased immune activity compared to the low-METTL5 patients using single-sample GSEA (ssGSEA) algorithm (**Figure 4B**). To explore the underlying molecular mechanisms of METTL5 in TNBC patients, we evaluated the biological function differing in the high-METTL5 and low-METTL5 subgroups of LUSC patients using gene set variation analysis (GSVA), which the results showed that high- METTL5 patients were mainly related with mitotic spindle, G2M checkpoint, E2F targets (**Figure 4C**), and cell cycle signaling pathway in the TCGA dataset (**Figure 4D**).

### Protein–protein interaction network and univariate cox regression analyses

**Figure 5:**
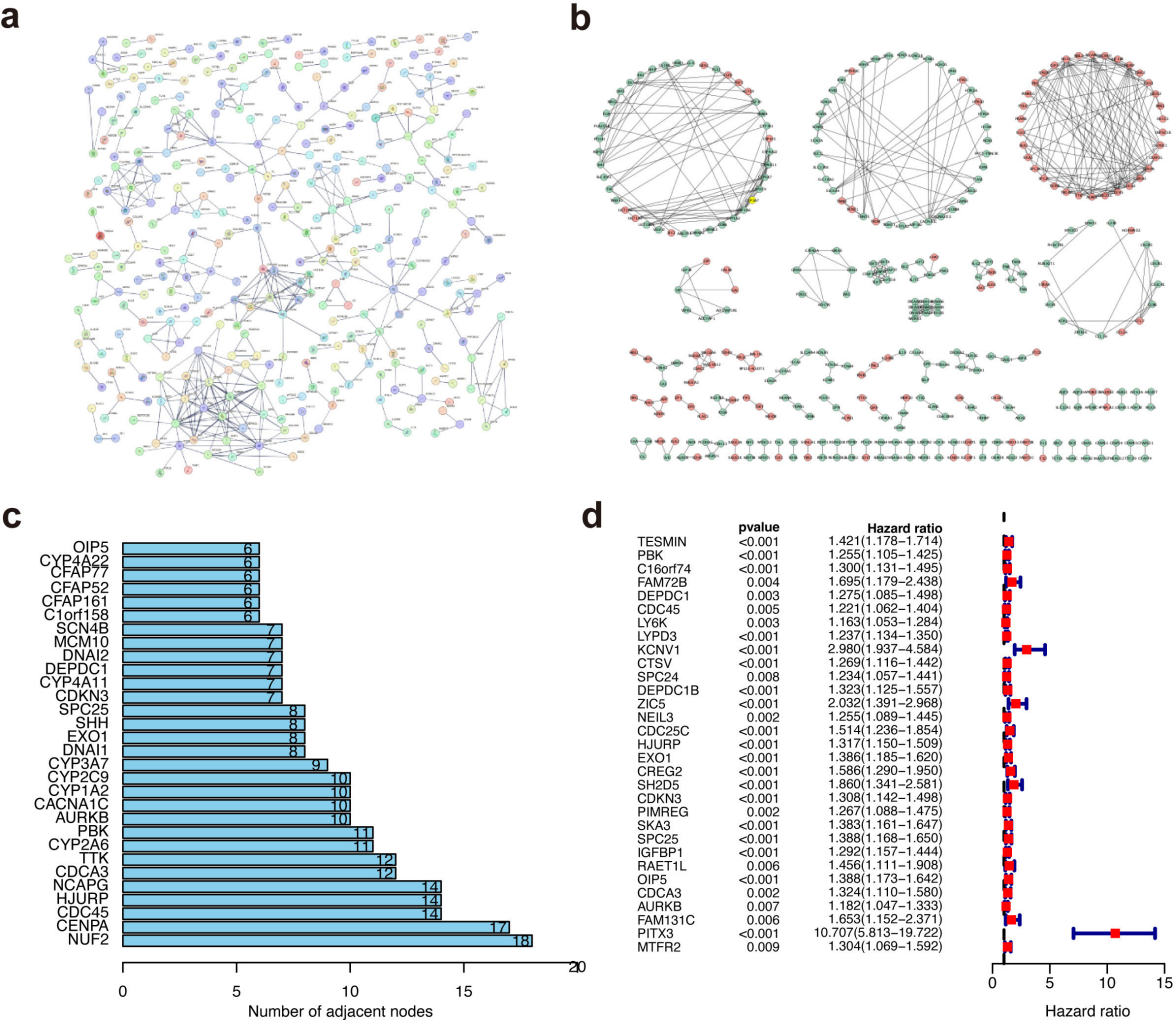
Protein–protein interaction network and univariate cox regression analyses. **(A)** The PPI network based on the STRING confidence score > 0.8. **(B)** The visualization of the PPI network. **(C)** The top 30 genes ordered by the number of nodes. **(D)** Univariate Cox analyses of DEGs.

The limma package was used to screened DEGs to investigate the potential biological behavior of METTL5 modification pattern between high-METTL5 and low-METTL5 expression groups. STRING database (confidence value > 0.8) was used to show the PPI network of the interactions among DEGs (**Figure 5A**), which were visualized in Cytoscape v3.8.2 (**Figure 5B**). Importantly, the top 30 genes were represented based on the number of nodes using bar plots, which may serve as hub nodes in the network (**Figure 5C**; **Supplementary Table S2**). Finally, a total 31 genes were of prognostic significance among the DEGs using univariate Cox regression analyses (**Figure 5D**; **Supplementary Table S3**).

### METTL5 promoted DEP domain containing 1 translation in an m6A‐dependent manner

**Figure 6:**
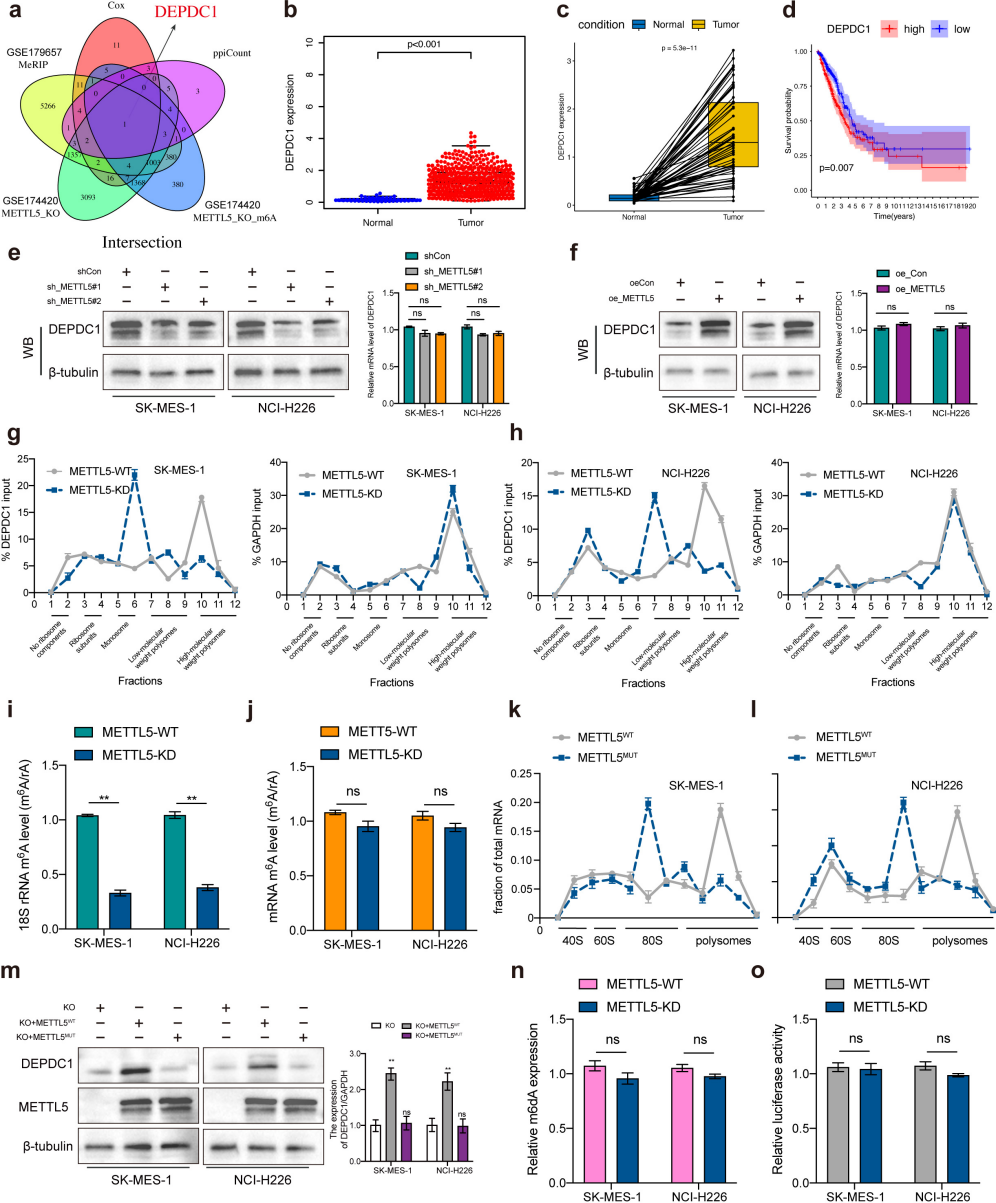
METTL5 promoted DEP domain containing 1 (DEPDC1) translation via 18S rRNA methyltransferase. **(A)** The Venn plot showed DEPDC1 was identified based on the intersection analysis. **(B, C)** The expression of DEPDC1 between normal and tumor tissues. **(D)** The survival analysis of LUSC patients with low and high DEPDC1 expression. **(E, F)** Relative DEPDC1 mRNA and protein expression levels in LUSC cells transected with METTL5 knockdown or overexpression. **(G, H)** The polysomes of METTL5‐WT and METTL5‐KD cells were extracted and subjected to a 10% to 50% sucrose gradient ultracentrifugation. The mRNA expression level in each fraction was determined by qRT‐PCR (upper) and visualized by DNA agarose gel (lower). **(I, J)** LC/MS was performed with an m6A antibody. **(K, L)** Polysome profiling by sucrose density gradient showing decreased polysomes and accumulated 80S monosomes. **(M)** Western blotting analysis of DEPDC1 protein levels in LUSC cells subjected to different treatments. **(N)** m6A DNA methylation assay detected alterations in m6dA of genomic DNA in METTL5-WT and METTL5-KD cells. **(O)** Dual luciferase reporter assay results indicated that METTL5 exhibits no regulatory effect on DNA methylation. n = 3 independent experiments, ns means no significant difference. Data are mean ± SEM. NS, non-significant, **p < 0.01, and ***p < 0.001. Two-tailed unpaired Student’s t test **(E–G, I, M–O)**.

The GEO data (GSE174420 and GSE179657) was employed to screen the METTL5 modified substrate genes with the cut-off criteria were set as | log2 fold change (FC) | < -0.5 and p < 0.05, and then DEPDC1 was identified through the intersection analysis of the 31 prognostic significance and the top 30 hub genes in the PPI network (**Figure 6A**). Subsequently, differential analysis showed DEPDC1 expression was significantly increased in LUSC comparing normal tissue using the TCGA dataset (**Figures 6B, C**). K-M analysis revealed that DEPDC1 overexpression in LUSC patients were poor survival (**Figure 6D**). Notedly, METTL5 knockdown or overexpression regulated DEPDC1 protein expression but did not significantly alter mRNA levels of DEPDC1 (**Figures 6E, F**). METTL5 regulates ribosome function by methylating 18S rRNA, resulting in changing indicated proteins levels (14). Then, polysome fractionation analysis was used to determine whether METTL5 had an impact on DEPDC1 translation in METTL5‐WT and METTL5‐KD cells, the results showed that METTL5 knockdown had no effect on the profile of GAPDH mRNA, whereas DEPDC1 mRNA was transferred from the heavier polysomal fractions to the lighter fractions (**Figures 6G, H**), which revealed that METTL5 regulated DEPDC1 mRNA translation. Therefore, we investigated whether DEPDC1 translational activity is dependent on METTL5 methylase activity. Importantly, LC/MS showed that METTL5 knockdown reduced the m6A level of 18S rRNA but had no effect on the m6A level of mRNA after separation of rRNA and mRNA (**Figures 6I, J**). Then, we constructed a METTL5 mutant without enzymatic activity (“NPPF” to “APPA”, Mut) (7), the results showed that METTL5 mutation decreased polysomes and accumulated 80S monosomes (**Figures 6K, L**), meanwhile overexpression of HA‐METTL5‐Mut remarkedly attenuated the expression of DEPDC1 protein compared to METTL5‐WT (**Figure 6M**). These results suggested that the translation of DEPDC1 is directly dependent on METTL5 18S rRNA methyltransferase activities. In addition, METTL3‐METTL14 complex, as a well‐known mRNA m6A methyltransferase, has been demonstrated to mediates DNA m6A methylation (m6dA) *in vitro (* 15). Notably, we were unable to find modification changes in the m6da region of genomic DNA following METTL5 knockdown (**Figure 6N**), which demonstrated that METTL5 was not involved in DNA methylation. Subsequently, the luciferase reporter assay determined that METTL5 had no effect on DEPDC1 promoter activity (**Figure 6O**). Taken together, these dates reveal that METTL5 was a pure RNA methylase and exhibited no regulatory effect on DNA methylation.

### METTL5 promotes LUSC progression through DEPDC1 expression

**Figure 7:**
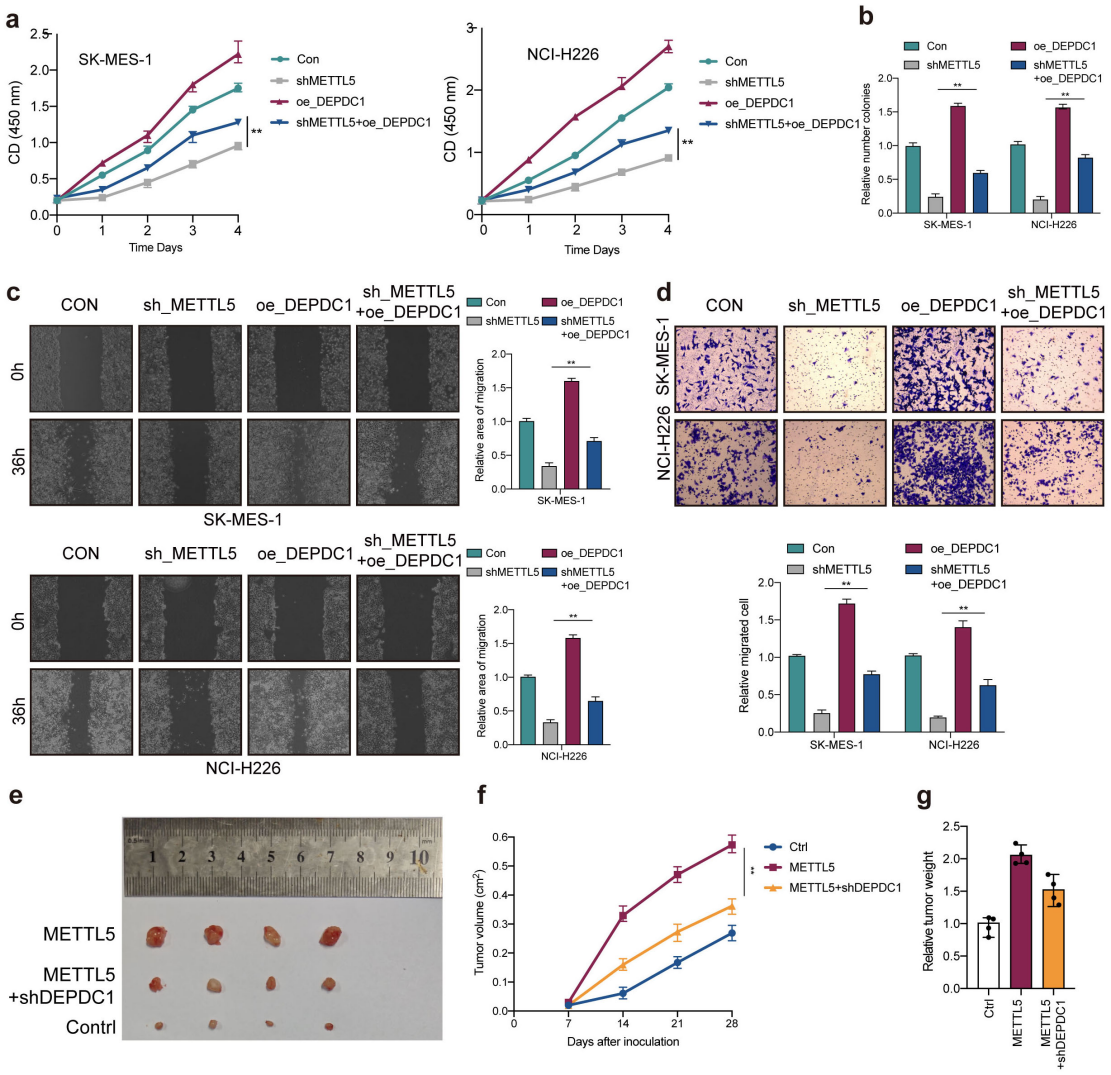
METTL5 promotes LUSC progression through DEPDC1 expression. **(A)** Cell viability was measured in METTL5 silencing cells with or without overexpression of DEPDC1. **(B)** Colony formation assay indicated the rescue effect of DEPDC1 on METTL5 silencing. **(C, D)** Transwell migration assays and wound healing assays were performed in METTL5-deficient cells with or without overexpression of DEPDC1. All the data are presented as the mean ± standard deviation (n=3). **P<0.01, compared with the control group. **(E)** The image of mice bearing subcutaneous tumors derived from SK-MES-1 cells treated with different treatment (Ctrl, METTL5 overexpression, or METTL5 overexpression+DEPDC1 knockdown) at the indicated times. **(F)** The xenograft growth curves for the METTL5 overexpression, METTL5 overexpression+ DEPDC1 knockdown, and Ctrl groups were plotted by measuring the tumor size (width2 × length × π/6) with a Vernier caliper every 7 days. **(G)** Nude mice were sacrificed, and xenografts were harvested and weighed.

Due to silencing METTL5 could reduce DEPDC1 expression. To verify the interaction between METTL5 and DEPDC1 in LUSC progression, rescue experiments were carried out. As expected, DEPDC1 overexpression could partially counteract the antitumor effects of MEETL5 knockdown on cell viability (**Figure 7A**), colony formation (**Figure 7B**) and migration (**Figures 7C, D**). In addition, overexpression of DEPDC1 also promoted cell proliferation, colony formation and migration in SK-MES-1 and NCI-H226 cells, confirming the oncogenic effects of DEPDC1 (**Figures 7A–D**). Importantly, in BALB/c mice’s model, MEETL5 overexpression-induced tumor progression could be partially abrogated by DEPDC1 knockdown (**Figures 7E–G**). Collectively, METTL5 promotes LUSC progression through DEPDC1 expression.

## Discussion

N6-methyladenosine (m6A) modification, as crucial regulators of LUSC progression, highlights the importance of understanding the crosstalk between these biological processes (16). Accumulating evidence has indicated that METTL5 is highly expressed and promotes tumor progression (6, 8, 14, 17), however, the role of METTL5 in LUSC is not explored.

In this study, among 26 m6A regulators (writers:10, erasers:3 and readers:13), we firstly identified the key m6A regulator METTL5 may be a crucial gene in LUSC using through CNV alteration frequency, differential analysis, correlation analysis, survival analysis, and qPCR essay. Then, we demonstrated that METTL5 was an independent prognostic factor in LUSC based on ROC curve, univariate analysis, and multivariate analysis. METTL5 has been confirmed to be overexpressed in a variety of tumors and affects patient prognosis, such as hepatocellular carcinoma (7, 18), Intrahepatic cholangiocarcinoma (ICC) (8), and breast cancer (14). We confirmed bioinformatically that the METTL5 expression was significantly higher in LUSC than in normal samples, and patients with high METTL5 expression had a worse prognosis. *In vitro* experiments, we demonstrated that METTL5 promotes tumor progression in LUSC via CCK-8, colony formation assay, transwell migration assay, and wound healing assay.

Next, we further explored the function of METTL5 in LUSC, the results showed older age, gender, and tumor stage were positive with METTL5 expression level. Surprisingly, high-METTL5 expression were no obvious immune activity compared to low-METTL5 patients. METTL5 is an enzyme that belongs to the S-adenosylmethionine (SAM)-dependent methyltransferase family (7). The main function of METTL5 is to regulate the stability, processing, and translation of RNA by methylating RNA molecules. Specifically, METTL5 adds methyl groups to certain positions of RNA (such as ribosomal RNA at the 5’ end), thereby affecting the structure and function of these RNAs, and thus playing an important role in the regulation of gene expression (14, 19). The role of METTL5 in cells may include: (1) rRNA methylation: One of the most well-known functions of METTL5 is to methylate specific positions of ribosomal RNA (rRNA), which is essential for the maturation and function of ribosomes. (2) Regulation of translation: By regulating the methylation state of rRNA, METTL5 may affect the efficiency of protein synthesis during translation. (3) Cell growth and proliferation: The activity of METTL5 is closely related to cell growth, proliferation, and response to environmental changes (20–22). Studies have found that METTL5 may be related to various biological processes such as cell stress response and cancer, so its function and regulatory mechanism are still one of the hot topics in current biomedical research. Our GSVA showed high-METTL5 patients were mainly related with G2M checkpoint, E2F targets, and cell cycle signaling pathway.

To further investigate the modification pattern of METTL5, DEGs are screened between high-METTL5 and low-METTL5 expression groups, and then subjected to the PPI network analysis, univariate Cox regression analyses, and differential analysis between METTL5 WT and METTL5 KO. Finally, screening DEPDC1 may be regulated by METTL5. DEPDC1 (DEP domain containing 1) is a protein that belongs to the DEP (Dishevelled, Egl-10, and Pleckstrin) domain family. The function of DEPDC1 is still under investigation, but existing studies have shown that it plays an important role in the occurrence, development, and metastasis of various cancers (23, 24). DEPDC1 is not only an important target for cancer biology research, but also provides potential clinical application prospects for early diagnosis and targeted treatment of cancer. Recent studies revealed that DEPDC1 promoted NSCLC development via TGF-β signaling pathway (11). DEPDC1 also play an essential role in the growth of bladder cancer cells (9). We confirmed bioinformatically that DEPDC1 was significantly upregulated in LUSC comparing normal tissue, and DEPDC1 overexpression showed poor survival. Notably, our results demonstrated that translation of DEPDC1 is directly dependent on METTL5 18S rRNA methyltransferase activities. *In vitro* experiments, rescue experiments were carried out to verify METTL5 promotes LUSC progression through enhancing DEPDC1 translation. Above data suggested METTL5-DEPDC1 axis has an important role in LUSC progression and provides a novel potential prognostic biomarker for LUSC.

## Conclusion

In summary, we demonstrated that the m6A writer METTL5 contributes to the tumorigenesis and poor prognosis of LUSC by enhancing DEPDC1 translation, providing a distinct mechanistic insight in m6A‐dependent, which should be helpful for developing DEPDC1 signaling‐targeted inhibitors.

## Data Availability

The original contributions presented in the study are included in the article/**Supplementary Material**. Further inquiries can be directed to the corresponding author.
